# Electricity in Surgery

**Published:** 1882-06

**Authors:** 


Electricity in Surgery. By John Butler, M. D. pp. 110. Price $1.00. Bœricke & Tafel: New York and Philadelphia. 1882.

Dr. Butler has for years made a specialty of electropathy and, therefore, in writing upon the subject, knows well whereof he speaks. In this work he gives a clear, though necessarily brief account of the many surgical operations wherein electricity can be used to advantage. The book is surgical in tone and like most of such works usurps fields of action that can be better worked with homœopathic therapeutics. With this exception the book is both interesting and valuable.

